# Association Between Body Image Before and During Pregnancy and Gestational Weight Gain in Japanese Women: A Prospective Cohort Study

**DOI:** 10.1007/s10995-023-03854-7

**Published:** 2023-12-05

**Authors:** Mie Shiraishi, Yuki Kurashima, Rio Harada

**Affiliations:** Department of Children and Women’s Health, Division of Health Sciences, Graduate School of Medicine, Osaka University, 1-7, Yamadaoka, Suita, 565-0871 Osaka Japan

**Keywords:** Body dissatisfaction, Body shape perception, Dietary restriction, Pregnancy, Weight gain

## Abstract

**Objectives:**

More than half of women do not achieve appropriate gestational weight gain. Maternal body image may be an important factor associated with gestational weight gain. However, this association has not been thoroughly evaluated. We aimed to elucidate whether body image parameters before and during pregnancy are associated with gestational weight gain in Japanese women.

**Methods:**

This prospective cohort study was conducted at a hospital in Osaka, Japan from March 2020 to March 2021. We recruited women with singleton pregnancies in their second and third trimesters. Body image was assessed using the Pregnancy and Weight Gain Attitude Scale and additional questions. Gestational weight gain was classified as insufficient, appropriate, or excessive based on recommended ranges in Japan. One-way analysis of variance, chi-square tests, and multinomial logistic regression analyses were performed to identify factors associated with insufficient or excessive weight gain.

**Results:**

Of 266 enrolled women, 47 had insufficient weight gain and 100 had excessive weight gain during pregnancy. Risk factors for excessive gestational weight gain included a history of dietary restriction before pregnancy, negative attitudes toward gestational weight gain, and perception of body shape as fat and body shape dissatisfaction during pregnancy. Perception of body shape as thin during pregnancy was identified as a risk factor for insufficient gestational weight gain.

**Conclusions:**

Body image before and during pregnancy may be an important factor in preventing insufficient or excessive gestational weight gain in Japanese women. Healthcare professionals should consider body image when providing health guidance on weight management to pregnant women.

## Significance

*What is Already Known?* Body image is a possible factor in gestational weight gain. Compared to women in other countries, Japanese women of reproductive age tend to have a negative body image.

*What this Study adds?* A history of dietary restriction before pregnancy and negative body image during pregnancy were associated with excessive gestational weight gain among Japanese women. Perception of body shape during pregnancy as thin was associated with insufficient gestational weight gain. Thus, even in a Japanese population, characterized by a majority of women with pre-pregnancy underweight or normal body mass index status, associations between negative body image and gestational weight gain were similar to those in other countries.

## Introduction

Recommended values for gestational weight gain are set according to the pre-pregnancy body mass index (BMI) to prevent small or large for gestational age and pregnancy complications such as gestational diabetes mellitus and hypertensive disorders of pregnancy (Hedderson et al., 2010; Goldstein et al., 2017; Uchinuma et al., 2021). However, more than half of women do not achieve appropriate weight gain during pregnancy (Herring et al., 2008; Andrews et al., 2018; Awn et al., 2021; Feng et al., 2021). In Japan, approximately 20–30% of women have insufficient gestational weight gain (Awn et al., 2021; Jin et al., 2021), and in recent years, insufficient gestational weight gain has received significant clinical attention due to the high incidence of low birth weight and small for gestational age (Harita et al., 2012; Uchinuma et al., 2021).

Weight control may be difficult during pregnancy because of physical symptoms, appetite changes, and food cravings, leading to insufficient or excessive weight gain (Harita et al., 2012; Feng et al., 2021). Furthermore, maternal body image before and during pregnancy has recently been shown to be a potentially important psychological factor related to gestational weight gain (Herring et al., 2008; Mehta et al., 2011; Bagheri et al., 2013; Andrews et al., 2018; Feng et al., 2021). Body image includes multidimensional self-perceptions and attitudes toward one’s outer appearance (Cash & Pruzinsky, 1990), and body image concepts related to body shape include perception and satisfaction with body shape, and negative attitudes toward gestational weight gain. These body image factors may influence diet and physical activity, which in turn may affect the degree of weight gain during pregnancy (Hill et al., 2013).

In Western countries, associations between body image before and during pregnancy and insufficient or excessive weight gain during pregnancy have been reported, with inconsistent results (Hayashi et al., 2006; Herring et al., 2008; Mehta et al., 2011; Bagheri et al., 2013; Mehta-Lee et al., 2013; Hill et al., 2013; Fealy et al., 2020; Feng et al., 2021; Schlaff et al., 2021). Several previous studies have indicated that women with normal BMI who overestimate their pre-pregnancy body shape, women with overweight or obese status who prefer a thinner body shape, and women with body shape dissatisfaction before or during pregnancy are more likely to experience excessive gestational weight gain (Herring et al., 2008; Bagheri et al., 2013; Fealy et al., 2020; Feng et al., 2021). However, other studies did not find an association between body image and gestational weight gain (Hill et al., 2013; Mehta-Lee et al., 2013; Schlaff et al., 2021). Thus, this association may depend on the mother’s background, such as the country of residence and actual body shape.

Japanese women of reproductive age tend to have a negative body image, including body shape overestimation and an excessive desire for thinness, because of the social tendency to consider slim as good (Hayashi et al., 2006). Because pre-pregnancy body image often persists throughout pregnancy (Duncombe et al., 2008), pregnant Japanese women as well as women of reproductive age may have a negative body image. In addition, women with negative body image are more likely to engage in dietary restriction (Uotani et al., 2020), and women who engage in dietary restriction before pregnancy are more prone to self-judged dietary restriction during pregnancy (Takimoto et al., 2011). Thus, negative body image before pregnancy may indirectly influence the degree of weight gain via dietary restriction during pregnancy.

In previous Japanese studies, some women with negative pre-pregnancy body image viewed body shape changes during pregnancy as a positive sign of fetal growth or natural changes during pregnancy (Hayashi et al., 2006; Tabata et al., 2015), while others became increasingly dissatisfied with their body shape as their pregnancy progressed (Maruyama et al., 2015). Additionally, the acceptability of body shape changes during pregnancy has been shown to be independent of pre-pregnancy BMI (Mizuguchi & Ebina, 2022). Furthermore, approximately 20% of Japanese women wish to avoid gaining too much weight during pregnancy in order to return to their pre-pregnancy weight soon after childbirth (Shiraishi et al., 2018). Thus, the way in which body image changes from pre-pregnancy to pregnancy varies across women, and these changes may be influenced by psychosocial background factors.

This study aimed to elucidate whether body image before and during pregnancy is associated with insufficient or excessive gestational weight gain in Japanese women. If a relationship exists, healthcare professionals should focus on body image when providing health guidance on weight management to pregnant women.

## Materials and Methods

### Study Design and Recruitment

This prospective cohort study was conducted at a perinatal medical center in Osaka, Japan, from March 2020 to March 2021. The survey was suspended from April to June 2020 because of the coronavirus disease 2019 (COVID-19) pandemic. Healthy Japanese women with singleton pregnancies were recruited at 20–26 weeks and 30–36 weeks of gestation. Those who had diabetes, hypertension, or psychological disorders; were aged less than 20 years; or had a low Japanese literacy level were excluded from the study. All participants received written and verbal information regarding the study protocol before providing written informed consent. The study protocol and procedure were approved by the ethics committees of our university and the survey institution (nos. 19,256 and 19–15, respectively).

Participants completed paper or online questionnaires while waiting for their pregnancy checkup. Paper questionnaires were used from March to April 2020, and online questionnaires were used after July 2020 to avoid contact between participants and researchers as much as possible, reducing the COVID-19 risk. Participants were asked to complete the online survey by accessing a URL or QR code. Participants who did not have sufficient time to complete the questionnaire in the hospital completed it after returning home and submitted it within two weeks. Missing or unclear data were resolved by mail. After the investigation, information on demographic variables and deliveries was obtained from medical charts.

A power analysis was conducted using G-Power version 3.1 (Faul et al., 2009). We estimated a total sample size of 308 women, with an odds ratio of 2.0 based on previous studies (Herring et al., 2008; Bagheri et al., 2013), a power of 0.80, and an alpha of 0.05 for logistic regression analysis. To account for potential dropouts, estimated at 20%, we aimed to recruit 385 women in total.

### Variables

Data on education level and physical and psychological variables related to pregnancy, such as unplanned pregnancy status, presence of nausea or vomiting at the time of the investigation, and body image, were collected using self-administered questionnaires.

#### Body Image Before Pregnancy

Variables related to pre-pregnancy body image included an excessive desire for thinness, perception of pre-pregnancy body shape, body shape satisfaction, and history of pre-pregnancy dietary restrictions. An excessive desire for thinness was assessed using the Japanese version of the Body Shape Silhouette (Nagasaka et al., 2008). Participants selected their desired pre-pregnancy body shape from nine silhouettes, which were scored from 1 to 9, with 1 being the slimmest silhouette and 9 being the heaviest silhouette. Each silhouette is assigned a BMI value according to the following equation: y = 13.888 + 1.654x (where y is the estimated BMI and x is the silhouette number) (Nagasaka et al., 2008). If the number obtained by subtracting the desired silhouette number from the silhouette number closest to the participant’s actual pre-pregnancy BMI was 2 or more, the participant was considered to have an excessive desire for thinness.

Perception of the pre-pregnancy body shape was assessed as too thin, thin, average, fat, or too fat using a 5-point Likert Scale. Participants were classified into three categories based on their response, with “too thin” or “thin” classified as “Thin”; “average” classified as “Average”; and “fat” or “too fat” classified as “Fat.” Misperception of the pre-pregnancy body shape was determined using the actual pre-pregnancy BMI. Perception of the pre-pregnancy body shape as larger or thinner than the actual body shape was classified as “overestimation” or “underestimation,” respectively.

Pre-pregnancy body shape satisfaction was assessed as very satisfied, satisfied, neither, dissatisfied, or very dissatisfied using a 5-point Likert scale. “Very satisfied” or “satisfied” was classified as “Satisfied”; “neither” was classified as “Neither”; and “dissatisfied” or “very dissatisfied” was classified as “Dissatisfied.”

#### Body Image During Pregnancy

Variables related to body image during pregnancy included perceptions of body shape during pregnancy, body shape satisfaction, attitudes toward gestational weight gain, and desire to return to pre-pregnancy weight soon after childbirth. Perception of body shape during pregnancy and body shape satisfaction were measured in the same manner as the corresponding pre-pregnancy variables.

Negative attitudes toward gestational weight gain were assessed using the Japanese version of the Pregnancy and Weight Gain Attitude Scale (J-PWGAS), which is a valid and reliable questionnaire for pregnant Japanese women (Kurashima et al., 2021). This scale was based on the scale developed by Palmer et al. (1985). The J-PWGAS comprises 17 items that require 5-point Likert scale responses ranging from “strongly agree” to “do not agree at all.” Average scores less than 3.0 points were considered to indicate a negative attitude toward gestational weight gain based on the original version of the PWGAS. The Cronbach’s α in the present study was 0.707.

### Demographic and Weight-Related Variables

Information on age, parity, pre-pregnancy weight and height, weight and gestational weeks at investigation, and weight and gestational weeks at delivery was obtained from medical charts. Pre-pregnancy BMI was calculated from the pre-pregnancy weight and height. Gestational weight gain was calculated by subtracting the pre-pregnancy weight from the weight at delivery.

Insufficient or excessive weight gain was determined using the recommendations of the Promotion Committee of the “Healthy Families (Sukoyaka Oyako 21)” Project (Japan Ministry of Health, Labour and Welfare, Promotion Council for Healthy Parents and Children 21, 2006). The recommended ranges of gestational weight gain according to pre-pregnancy weight status are 9–12 kg for those with underweight status, 7–12 kg for those with normal BMI, and approximately 5 kg for those with overweight or obese status. Thus, we defined insufficient gestational weight gain according to the pre-pregnancy weight status as less than 9 kg for those with underweight status, less than 7 kg for those with normal BMI, and less than 3 kg for those with overweight or obese status. We defined excessive gestational weight gain according to the pre-pregnancy weight status as more than 12 kg for those with underweight or normal BMI status, and more than 5 kg for those with overweight or obese status.

### Statistical Analysis

Summary statistics of demographic and clinical characteristics are expressed as frequencies and percentages for categorical variables and as means and the standard deviation (SD) for continuous variables after testing for normality. Differences in demographic and body image variables among gestational weight gain groups (insufficient, appropriate, and excessive) were evaluated using the one-way analysis of variance or chi-square test. In addition, multinomial logistic regression analyses were performed to identify body image factors associated with insufficient or excessive weight gain. Each body image variable was entered as an independent variable; to account for multicollinearity, each variable was analyzed separately. Variables with a *p*-value < 0.10 on binary analysis were entered as covariates into multinomial logistic regression models. A correlation matrix of all the predictors was checked to test for multicollinearity. Associations between body image factors before and during pregnancy and pre-pregnancy BMI were evaluated using the Fisher’s exact or chi-square test., Differences were considered statistically significant at a two-tailed *p*-value of < 0.05. All statistical analyses were conducted using the IBM Statistical Package for Social Sciences for Windows (version 24.0; IBM Japan, Tokyo, Japan).

## Findings

Of 364 healthy pregnant women invited to participate in the study, 316 (86.8%) provided written informed consent. Among these, 50 were excluded from analysis because of consent withdrawal (n = 14) or missing data (n = 36). Thus, data from 266 women (73.1%) were included in the final analysis. There were no differences in characteristics between participants and dropouts.

Table 1 summarizes the characteristics of the participants. The mean (SD) maternal age was 31.8 (6.1) years and mean (SD) pre-pregnancy BMI was 21.2 (3.2) kg/m^2^. Of the 266 women, 47 (17.7%) had insufficient weight gain, 119 (44.7%) had appropriate weight gain, and 100 (37.6%) had excessive weight gain during pregnancy. Women with excessive weight gain were significantly younger than those in the other groups (*p* = 0.048). Primiparas had significantly lower rates of appropriate weight gain during pregnancy than multiparas (*p* = 0.035).

**Table 1:** Participants characteristics

	All participants (n = 266)		Gestational weight gain						
			Insufficient (n = 47)		Appropriate (n = 119)		Excessive (n = 100)		*p*
	Mean ± SD or n (%)		Mean ± SD or n (%)		Mean ± SD or n (%)		Mean ± SD or n (%)		
Age [years]	31.8	± 6.1	32.5	± 6.0	32.5	± 6.0	30.6	± 6.0	0.048
Parity: Primipara [n (%)]	150	(56.4)	28	(59.6)	57	(47.9)	65	(65.0)	0.035^a^
Gestational age at investigation [weeks]	28.4	± 5.7	29.2	± 5.7	28.2	± 6.0	28.2	± 5.5	0.533
Education level [n (%)]									< 0.001^a^
University or above	178	(66.9)	34	(72.3)	93	(78.2)	51	(51.0)	
Other	88	(33.1)	13	(27.7)	26	(21.8)	49	(49.0)	
Height [cm]	158.7	± 5.3	158.7	± 5.2	158.8	± 4.8	158.6	± 6.0	0.982
Pre-pregnancy weight [kg]	53.5	± 8.4	51.9	± 7.9	52.0	± 7.0	56.0	± 9.6	0.001
Pre-pregnancy BMI [kg/m^2^]	21.2	± 3.2	20.6	± 3.3	20.6	± 2.4	22.3	± 3.7	< 0.001
Underweight (< 18.5 kg/m^2^)	46	(17.3)	13	(27.7)	18	(15.1)	15	(15.0)	< 0.001^a^
Normal BMI (18.5–24.9 kg/m^2^）	183	(68.8)	30	(63.8)	97	(81.5)	56	(56.0)	
Overweight and obesity (25.0–kg/m^2^)	37	(13.9)	4	(8.5)	4	(3.4)	29	(29.0)	
Unplanned pregnancy	46	(17.3)	8	(17.0)	22	(18.4)	16	(16.0)	0.301^a^
Nausea or vomiting at investigation ^b^	75	(32.9)	15	(39.5)	37	(33.6)	23	(28.8)	0.498^a^
Gestational age at delivery [weeks]	39.1	± 1.3	38.9	± 1.2	39.0	± 1.2	39.3	± 1.3	0.060
Gestational weight gain [kg]	10.8	± 4.4	5.4	± 2.4	9.6	± 1.6	14.6	± 3.9	< 0.001

*BMI* body mass index, *SD* standard deviation

One-way analysis of variance. ^a^Chi-square test, ^b^n = 228.

Differences in body image variables before and during pregnancy between gestational weight gain groups are shown in Table 2. Bivariate analyses of pre-pregnancy body image variables showed that perception of pre-pregnancy body shape as fat (p = 0.002), excessive desire for thinness (p = 0.001), and a history of dietary restriction (p < 0.001) were significantly associated with excessive gestational weight gain. Most pre-pregnancy body image variables were significantly correlated with each other (Table 3.). Bivariate analyses of body image variables during pregnancy showed that negative attitudes toward gestational weight gain (p = 0.001), perception of body shape as fat (p < 0.001) and body shape dissatisfaction during pregnancy (p < 0.001), and desire to return to pre-pregnancy weight soon after childbirth (p = 0.033) were significantly associated with excessive gestational weight gain. Pre-pregnancy body shape perception and body shape satisfaction were strongly associated with body shape perception and satisfaction during pregnancy and pre-pregnancy BMI (p < 0.001, Table 4).

**Table 2:** Body image before and during pregnancy according to gestational weight gain

	All participants (n =266)		Gestational weight gain						
			Insufficient (n = 47)		Appropriate (n = 119)		Excessive (n = 100)		*p*
	n (%) or Mean±SD		n (%) or Mean±SD		n (%) or Mean±SD		n (%) or Mean±SD		
*Perception of pre-pregnancy body shape* [n (%)]									0.002^a^
Thin	24	(9.0)	10	(21.3)	9	(7.6)	5	(5.0)	
Average	124	(46.6)	24	(51.1)	60	(50.4)	40	(40.0)	
Fat	118	(44.4)	13	(27.7)	50	(42.0)	55	(50.0)	
*Misperception of pre-pregnancy body shape* [n (%)]									0.322^a^
Underestimation	12	(4.5)	4	(8.5)	4	(3.4)	4	(4.0)	
Appropriate-estimation	144	(54.1)	26	(55.3)	59	(49.6)	59	(59.0)	
Overestimation	110	(41.4)	17	(36.2)	56	(47.1)	37	(37.0)	
*Pre-pregnancy body shape satisfaction* [n (%)]									0.256
Satisfied	79	(29.7)	15	(31.9)	33	(27.7)	31	(31.0)	
Dissatisfied	122	(45.9)	19	(40.4)	51	(42.9)	52	(52.0)	
Neither	65	(24.4)	13	(27.7)	35	(29.4)	17	(17.0)	
Excessive desire for thinness before pregnancy [n (%)]	75	(28.2)	6	(12.8)	29	(24.4)	40	(40.0)	0.001
History of dietary restriction before pregnancy [n (%)]	82	(30.8)	8	(17.0)	28	(23.5)	46	(46.0)	<0.001
Pregnancy weight gain attitude scale [score]	3.08	± 0.46	3.21	± 0.46	3.09	± 0.47	3.01	± 0.44	0.041^b^
Negative attitudes toward gestational weight gain [n (%)]	108	(40.6)	11	(23.4)	43	(36.1)	54	(54.0)	0.001
*Perception of body shape during pregnancy* [n (%)]									<0.001^a^
Thin	7	(2.6)	6	(12.8)	1	(0.8)	0	(0.0).	
Average	105	(39.5)	29	(61.7)	58	(48.7)	18	(18.0)	
Fat	154	(57.9)	12	(25.5)	60	(50.4)	82	(82.0)	
*Body shape satisfaction during pregnancy* [n (%)]									<0.001
Satisfied	63	(23.7)	13	(27.7)	34	(28.6)	16	(16.0)	
Dissatisfied	110	(41.4)	10	(21.3)	37	(31.1)	63	(63.0)	
Neither	93	(35.0)	24	(51.1)	48	(40.3)	21	(21.0)	
Desire to return to their pre-pregnancy weight soon after childbirth [n (%)]	135	(50.8)	20	(42.6)	54	(45.4)	61	(61.0)	0.033

*SD* standard deviation

Chi-square test, ^a^Fisher's exact test, ^b^One-way analysis of variance.

**Table 3:** Correlations between body image variables before and during pregnancy

	1	2	3	4	5	6	7	8
1. Perception of pre-pregnancy body shape ^a^	1							
2. Misperception of pre-pregnancy body shape ^b^	0.594^***^	1						
3. Body shape satisfaction before pregnancy ^c^	0.540^***^	0.231^***^	1					
4. Excessive desire for thinness before pregnancy ^d^	0.404^***^	0.092	0.258^***^	1				
5. History of dietary restriction before pregnancy ^d^	0.161^**^	0.033	0.061	0.197^**^	1			
6. Negative attitudes toward gestational weight gain ^d^	0.152^*^	0.195^**^	0.079	0.162^**^	0.227^***^	1		
7. Perception of body shape during pregnancy ^a^	0.413^***^	0.209^**^	0.224^***^	0.252^***^	0.287^***^	0.340^***^	1	
8. Body shape satisfaction during pregnancy ^c^	0.302^***^	0.099	0.308^***^	0.259^***^	0.217^***^	0.410^***^	0.573^***^	1
9. Desire to return to their pre-pregnancy weight soon after childbirth ^d^	0.080	-0.008	0.099	0.082	0.137^*^	0.324^***^	0.273^***^	0.343^***^

Spearman's rank correlation coefficient. ^*^p<0.05, ^**^p<0.01, ^***^p<0.001

^a^1: Thin, 2: Average, 3: Fat

^b^1: Underestimation, 2: Appropriate-estimation, 3: Overestimation

^c^1: Satisfied, 2: Neither, 3: Dissatisfied

^d^1: Yes, 0: No

**Table 4:** Correlations between body image variables before and during pregnancy and pre-pregnancy BMI (n=266)

	Perception of body shape during pregnancy							Pre-pregnancy BMI						
	Thin		Average		Fat		*p*^a^	Underweight		Normal BMI		Overweight or obese		*p*^a^
	n	(%)	n	(%)	n	(%)		n	(%)	n	(%)	n	(%)	
Perception of pre-pregnancy body shape							<0.001							<0.001
Thin	4	(16.7)	13	(54.2)	7	(29.2)		14	(58.3)	9	(37.5)	1	(2.7)	
Average	3	(2.4)	68	(54.8)	53	(42.7)		26	(21.0)	96	(77.4)	2	(1.6)	
Fat	0	(0.0)	24	(20.3)	94	(79.7)		6	(5.1)	78	(66.1)	34	(28.8)	
	Body shape satisfaction during pregnancy							Pre-pregnancy BMI						
	Satisfied		Dissatisfied		Neither		*p*^b^	Underweight		Normal BMI		Overweight or obese		*p*^b^
	n	(%)	n	(%)	n	(%)		n	(%)	n	(%)	n	(%)	
Pre-pregnancy body shape satisfaction							<0.001							<0.001
Satisfied	31	(39.2)	24	(30.4)	24	(30.4)		26	(32.9)	48	(60.8)	5	(6.3)	
Dissatisfied	15	(12.3)	70	(57.4)	37	(30.3)		6	(4.9)	87	(71.3)	29	(23.8)	
Neither	17	(26.2)	16	(24.6)	32	(49.2)		14	(21.5)	48	(73.8)	3	(4.6)	
BMI: body mass index, ^a^Fisher's exact test, ^b^Chi-square test.														

Multinomial logistic regression analyses revealed that the following factors were associated with a higher risk of excessive weight gain during pregnancy: a history of dietary restriction before pregnancy, negative attitudes toward gestational weight gain, perception of body shape during pregnancy as fat, and body shape dissatisfaction during pregnancy (Table 5). In addition, perception of body shape during pregnancy as thin was significantly associated with a higher risk of insufficient weight gain, and perception of body shape during pregnancy as fat was associated with a lower risk of insufficient weight gain.

**Table 5:** Associations between body image variables before and during pregnancy and gestational weight gain (n=266)

	Insufficient weight gain (vs Appropriate)					Excessive weight gain (vs Appropriate)				
	B	SE	AOR	(95% CI)	*p*	B	SE	AOR	(95% CI)	*p*
*Body image before pregnancy*										
*Perception of pre-pregnancy body shape*^a^										
Thin (Ref. Average)	1.046	0.547	2.847	(0.974–8.323)	0.056	− 0.089	0.638	0.915	(0.262–3.196)	0.890
Fat (Ref. Average)	− 0.496	0.426	0.609	(0.264–1.405)	0.245	0.183	0.332	1.200	(0.626–2.301)	0.583
*Misperception of pre-pregnancy body shape*^a^										
Underestimation (Ref. Appropriate)	0.839	0.758	2.314	(0.524–10.220)	0.268	− 0.630	0.812	0.533	(0.109–2.615)	0.438
Overestimation (Ref. Appropriate)	− 0.562	0.394	0.570	(0.264–1.234)	0.154	− 0.079	0.332	0.924	(0.482–1.771)	0.811
*Body shape satisfaction before pregnancy*^a^										
Dissatisfied (Ref. Satisfied)	− 0.107	0.452	0.899	(0.370–2.180)	0.813	− 0.270	0.376	0.763	(0.365–1.595)	0.473
Neither (Ref. Satisfied)	− 0.183	0.462	0.833	(0.337–2.061)	0.693	− 0.717	0.422	0.488	(0.213–1.117)	0.090
Excessive desire for thinness before pregnancy^a^	− 0.756	0.503	0.469	(0.175–1.257)	0.132	0.447	0.335	1.563	(0.811–3.011)	0.182
History of dietary restriction before pregnancy^a^	− 0.450	0.455	0.638	(0.261–1.556)	0.323	0.750	0.324	2.117	(1.123–3.993)	0.020
*Body image during pregnancy*										
Negative attitudes toward gestational weight gain^a^	− 0.688	0.402	0.503	(0.229–1.105)	0.087	0.853	0.305	2.348	(1.290–4.273)	0.005
*Perception of body shape during pregnancy*^a^										
Thin (Ref. Average)	2.482	1.120	11.971	(1.332–107.60)	0.027					
Fat (Ref. Average)	− 0.981	0.403	0.375	(0.170–0.826)	0.015	1.307	0.344	3.694	(1.883–7.247)	<0.001
*Body shape satisfaction during pregnancy*^a^										
Dissatisfied (Ref. Satisfied)	− 0.353	0.491	0.703	(0.268–1.839)	0.472	1.145	0.400	3.142	(1.435–6.879)	0.004
Neither (Ref. Satisfied)	0.187	0.418	1.205	(0.531–2.734)	0.655	− 0.290	0.432	0.748	(0.321–1.744)	0.491
Desire to return to pre-pregnancy weight soon after childbirth^a^	− 0.137	0.355	0.872	(0.435–1.748)	0.699	0.529	0.303	1.697	(0.936–3.075)	0.081

*AOR* adjusted odds ratio, *CI* confidence interval, *SE* standard error

Multinomial logistic regression analysis. Separate analyses were conducted for each variable related to body image. Nagelkerke R^2^= 0.20–0.35

Dependent variable: Insufficient (n=47), Appropriate (n=119), Excessive (n=100)

^a^Adjusted for age, parity, education levels, pre-pregnancy body mass index, and gestational age at delivery

## Discussion

The present study demonstrated that a history of dietary restriction before pregnancy and negative body image during pregnancy are associated with a higher risk of excessive gestational weight gain in Japanese women. In addition, perception of body shape during pregnancy as thin was identified as a factor related to insufficient gestational weight gain.

Among pre-pregnancy body image variables, only a history of dietary restriction was associated with excessive gestational weight gain. Pre-pregnancy body shape perception and satisfaction, and excessive desire for thinness were not associated with gestational weight gain; these results differ from those of previous studies conducted in Western countries (Herring et al., 2008; Mehta et al., 2011; Bagheri et al., 2013). This difference in results could be related to differences in the strength of negative body images (Watson et al., 2016). The three above-mentioned variables indicate attitudes toward body shape, whereas a history of dietary restriction before pregnancy indicates behaviors related to body image. Negative attitudes toward body shape do not necessarily lead to weight loss behaviors (Mase et al., 2015; Mori et al., 2016); thus, women who underwent dietary restrictions to lose weight may be presumed to have stronger negative attitudes toward body shape.

A previous Japanese cross-sectional study of 248 pregnant women indicated that women who restricted their diet before pregnancy were at a higher risk for self-judged dietary restriction during pregnancy (Takimoto et al., 2011). Therefore, we hypothesized that women with a history of dietary restriction before pregnancy would be more likely to have insufficient gestational weight gain. However, contrary to our expectations, the results indicated that a history of dietary restriction was associated with an increased risk of excessive weight gain. Studies conducted in Western countries have reported that women who undergo dietary restriction before pregnancy are less likely to do so during pregnancy, and tend to eat more during pregnancy (Clark & Ogden, 1999; Tutkuviene et al., 2018). This may be related to a reduction in the pressure to obtain a desired body shape during pregnancy, freeing women from stigma and dieting (Fox & Yamaguchi, 1997). Furthermore, negative attitudes toward body shape before pregnancy often turn into positive attitudes during pregnancy because of the unique situation in which body shape changes reflect fetal growth. Accordingly, this relief from body shape pressures during pregnancy may lead to excessive gestational weight gain through increased dietary intake. From another perspective, women who controlled their weight by adjusting their diet and physical activity before pregnancy may have difficulty controlling their weight under the special circumstances of pregnancy, where appetite and food cravings change (Fox & Yamaguchi, 1997) and strict dietary restrictions and vigorous exercise are not possible. Thus, for women who restricted their diet before pregnancy, healthcare professionals may need to understand their attitudes toward weight gain and weight control behaviors (Takimoto et al., 2011) and suggest lifestyle adjustments to prevent excessive weight gain during pregnancy.

Previous studies conducted in Western countries have reported conflicting results regarding whether pregnant women who perceive themselves as fat gain less weight or more weight during pregnancy (Hill et al., 2013; Andrews et al., 2018). In the present study, women who perceived themselves as fat had an increased risk of excessive gestational weight gain, similar to the findings of Andrews et al. (2018). Since 80% of our participants who perceived themselves as fat during pregnancy had underweight or normal BMI status before pregnancy, we assume that they had less pressure from healthcare professionals to prevent unnecessary weight gain and, therefore, less motivation to suppress their weight gain. This could be one of the reasons for the observed increased risk of excessive gestational weight gain, among women who perceived themselves as fat. Alternatively, these participants may have perceived themselves as fat because they had already exceeded or were likely to exceed the recommended weight gain range at the time of the survey.

The present study also indicated that negative attitudes toward gestational weight gain and body shape dissatisfaction during pregnancy were significantly associated with excessive gestational weight gain, although previous studies have reported contradictory results (Copper et al., 1995; Heery et al., 2016). These associations may also be related to a negative body image caused by already exceeding one’s expected weight gain and difficulty in adjusting one’s diet due to changes in appetite and other factors, as mentioned above. Causal associations were unclear in this study. However, the results suggest that healthcare professionals may need to focus on women with these characteristics as a high-risk population for excessive weight gain during pregnancy.

Only the perception of body shape during pregnancy as thin was associated with a higher risk of insufficient gestational weight gain. This perception may derive from little change in weight from pre-pregnancy levels due to pregnancy-related nausea and other physical reasons. Another possibility is that women who perceived their body shape during pregnancy as thin were controlling their weight to avoid gaining more than what they considered as acceptable. The target weight gain during pregnancy for some Japanese women is lower than the recommended range (Ogawa et al., 2018). In the present study, we did not investigate the effect of target weight gain on body shape perception and insufficient gestational weight gain, and the small number of pregnant women who perceived themselves as thin precluded a clear reason for these associations. Further studies are needed to confirm this association.

Body image varies according to country and socio-cultural background. According to previous studies, Japanese women are likely to overestimate their own body shape and have an excessive desire for thinness, even when they have normal BMI or underweight status (Hayashi et al., 2006). Additionally, the prevalence of women with negative body image is higher in Japan than in other countries (Wardle et al., 2006). These Japanese trends may have influenced the present results regarding associations between body image and gestational weight gain, which may differ from those in other countries. In addition, although the actual BMI could be a confounding factor in these associations, we were unable to examine its impact due to the small sample size. Therefore, more detailed study that considers the actual BMI is required.

To our knowledge, this study is the first to identify associations between various body image parameters and weight gain during pregnancy in a Japanese population. Even in this population, which is characterized by a majority of women with pre-pregnancy underweight or normal BMI status, the observed association between negative body image and gestational weight gain was similar to that in other countries. However, the present study has several limitations. First, recall bias may have affected the results because the perception of the pre-pregnancy body shape was assessed during pregnancy using recall methods. Second, the pre-pregnancy body weight was self-reported and may have differed from the actual weight. Nevertheless, we speculated that such differences would be small because a strong correlation between self-reported and measured weight has been previously reported in Japanese women (Okamoto et al., 2017). Third, some variables related to body image could not be measured using a scale because there was no validated scale in Japan. Fourth, the final sample size was smaller than planned. This might have limited the statistical power of the study. Fifth, generalizations of the results should be made with caution because the research data were obtained from a single facility in Osaka. However, the mean age, education level, and gestational weight gain of our participants were similar to those in the national data (Ministry of Health, Labour and Welfare of Japan, 2019). Therefore, we suggest that our results are applicable to healthy Japanese women with singleton pregnancies.

## Conclusions

The present study indicated that a history of dietary restriction before pregnancy and body image factors during pregnancy, including the perception of body shape as fat, body shape dissatisfaction, and negative attitudes toward gestational weight gain are associated with excessive gestational weight gain in Japanese women. In addition, the perception of body shape during pregnancy as thin was associated with insufficient gestational weight gain. Healthcare professionals should consider these body image factors when providing health guidance on weight management during pregnancy. However, further larger-scale cohort studies are needed to confirm the associations between body image parameters and gestation weight gain because this study had a small sample size and was conducted at a single institution.

## Data Availability

The data underlying this article will be shared on reasonable request to the corresponding author.
